# News and Notes

**Published:** 1908-03

**Authors:** 


### NEWS AND NOTES

Wm. Green, Granite Falls, Wash., is said to have been fined $50 January 2nd for practicing medicine without license.

The Canadian Medical Association will hold its annual meeting this year in Ottawa, June 9 to 11.

Little Rock Conference will begin at once a movement to erect a Methodist hospital in Arkansas to cost not less than $200,000.

According to the annual report of the State Commission in Lunacy, the total number of committed insane in the State of New York on October 1, 1907, was 29,093—13,927 men and 15,166 women.

The prominent members of the profession and the public authorities at Berlin tendered a banquet to Albert Neisser, January 31, on his return from his research on syphilis in Java.

Members of the Georgia Medical Association are urged to send in at once the titles of the papers they are to read at the Fitzgerald meeting in April.

The officers of Mt. Sinai Hospital announce the receipt of a gift of $200,000 from Dr. Adolph Lewisohn, which is to be added to the endowment fund of the hospital.

A new international quarterly, to be called Epilepsia, is to be devoted to the study and treatment of this disease from the therapeutic, social and judicial points of view.

---

If the plans of Bishop C. K. Nelson are consummated, Atlanta is to have another hospital and two colleges; all of which are to be under the auspices of the Episcopal church.

---

The Louisiana State Board of Medical Examiners announce that henceforth they will require proof of identification of all applicants for license who are not personally known to some member of the Board.

---

The Richmond board of health recently held a tuberculosis exhibit, which was attended by 11,517 people of Richmond and vicinity. More than fifty speakers discussed tuberculosis at the different meetings held during the week of the exhibit.

---

The chief of the bureau of medicine and surgery, U. S. Navy, has asked the House Committee on Naval Affairs to recommend an appropriation of $1,000,000 for the construction and equipment of a hospital ship for the navy.

---

Dengue was discussed at the last meeting of the Galveston Medical Association, and the conclusion drawn that contagion is carried by the mosquito in precisely the same way as yellow fever. All agreed on this and a resolution, requesting the County Commissioners to make an appropriation for the extermination of the mosquitoes, was adopted.

---

A Western Journal considers that some medical societies "are hewing to the line pretty close in their endeavor to uphold ethics." At Peoria, in Illinois, a member was hauled over the coals recently for driving a piebald horse on the ground that it was a bid for public attention.

---

Dr. James P. Rosser, for a long time a practicing physician in Atlanta and well known throughout the northern section of the State, having been born and reared in Walton county, where he was engaged in his profession for years, died February 10th at his home, 351 Cherokee Avenue, of pneumonia, at the age of 71, after a short illness.

---

The United States Civil Service Commission announces an examination on March 4, 1908, at the places mentioned in the list printed hereon, to secure eligibles from which to make certification to fill a vacancy in the position of acting assistant surgeon, Public Health and Marine-Hospital Service, for duty at St. John's River Quarantine Station, Mayport, Fla., at $125 per month, and vacancies requiring similar qualifications as they may occur.

---

An exhibit illustrating the congestion of population in New York will be held in the American Museum of Natural History for a period of two weeks, beginning Monday, March 9th. On the opening night Governor Hughes, Mayor McClellan, the Italian Ambassador to the United States, and several of the commissioners of New York city will speak, and on the three following days there will be a general discussion of the various aspects of the congestion of population.

---

Dr. B. W. Allen was elected president of the Muscogee County Medical Society. Other officers elected were: Dr. W. T. Gautier, vice-president; Dr. T. E. Mitchell, treasurer; Dr. Martin Crook, secretary; Dr. H. S. Monroe, censor, and Dr. C. A. Dexter, delegate to the State Convention.

The suit brought by Professor Adamkiewicz, formery professor at Warsaw, against the firm of E. Merck, of Darmstadt, claiming damages for non-fulfillment of contract in regard to pushing the sale of his "cancroin" for the cure of cancer, was lost, and the final court of appeals has confirmed the judgment of the lower court, based on expert testimony as to the worthlessness of the alleged remedy.

---

Dr. G. F. Brigham, aged 62, lost his life in Girard, Ga., January 30, while entering a burning building to recover his medicine case and some valuable papers. Eight stores were destroyed by the fire, which originated in the home of Dr. F. A. Buxton. Dr. Brigham being caught in the falling timbers was burned to death. He leaves a wife and six children.

---

The superintendent of Johns Hopkins Hospital reports that during 1907 there were 4,576 patients in the hospital, 2,137 males and 2,439 females. At the close of the year there were 231 white patients and 72 colored patients in the hospital. There were 4,859 persons treated, of whom 3,779 were white and 1,080 colored; 1,466 were medical cases, 1,685 surgical, 986 gynecologic and 439 obstetric.

---

The tenth annual meeting of the Tri-State Medical Association of the Carolinas and Virginia will be held in Charlotte, February 18 and 19, under the presidency of Dr. Stuart McGuire, Richmond. Dr. J. Howell Way, Waynesville, is secretary. Headquarters will be at the Selwyn Hotel, and the topic for the general debate will be "The Treatment of Epilepsy."

---

The Archives of Diagnosis is the title of a new quarterly journal, edited by Henrich Stern, of New York. The first issue, just published, makes an excellent showing and contains 116 pages of first-class matter composed of original articles and abstracts on the diagnosis of pathologic conditions. The quality of the subject matter, and the care with which it is edited, reflects credit upon Dr. Stern, who is already favorably known to the medical world. We extend our best wishes to the Journal, and predict for it success, in elucidating this important part of medicine.

### PIEDMONT SANITORIUM INCREASES STAFF.

The Piedmont Sanatorium which has become favorably known throughout the South, and which heretofore has only taken care of the patients of Dr. Floyd McRae and Dr. Amster, has adopted a wider scope for its activity. It is a well merited compliment to the institution, that the following prominent men are now on its staff: Dr. Floyd W. McRae, General surgery and gynscolocgy; Dr. Wm. Perrin Nicholson, general surgery; Dr. Michael Hoke, orthopedic surgery; Dr. L. Sage Hardin, rectal diseases; Dr. E. Bates Block, nervous diseases; Dr. L. Amster, gastro-intestinal diseases; Dr. Phinizy Calhoun, eye, ear, nose and throat; Dr. Marion McH. Hull, general medicine; Dr. James E. Paulin, Pathologist; Dr. Annie L. Sawyer, obstetrics.

### ARMY MEDICAL CORPS EXAMINATIONS.

Preliminary examinations for appointment of Assistant Surgeons in the army will be held on May 4th and August 3d, 1908, at points to be hereafter designated.

Full information concerning the examination can be procured upon application to the Surgeon General, U. S. Army, Washington, D. C. The essential requirements to securing an invitation are that the applicant shall be a citizen of the United States, shall be between twenty-two and thirty years of age, a graduate of a medical school legally authorized to confer the degree of doctor of medicine, shall be of good moral character and habits, and shall have had at least one year's hospital training or its equivalent in practice. The examinations will be held concurrently throughout the country at points where boards can be convened. Due consideration will be given to the localities from which applicants are received, in order to lessen the traveling expenses of applicants as much as possible.

Applicants holding diplomas from reputable literary or scientific colleges, normal schools or high schools, or graduates of medical schools which require an entrance examination satisfactory to the faculty of the Army Medical School, will not be examined in subjects of general preliminary education.

In order to perfect all necessary arrangements for the examinations of May 4th, applications must be complete and in possession of the Surgeon General on or before April 1st. Early attention is therefore enjoined upon all intending applicants.

There are at present twenty-three vacancies in the Medical Corps of the Army.

### SMITHSONIAN INSTITUTION.

### Hodgkins Fund Prize.

In October, 1891, Thomas George Hodgkins, Esquire, of Setauket, New York, made a donation to the Smithsonian Institution, the income from a part of which was devoted to "the increase and diffusion of more exact knowledge in regard to the nature and properties of atmospheric air in connection with the welfare of man."

In the furtherance of the donor's wishes, the Smithsonian Institution has from time to time offered prizes, awarded medals, made grants of investigations, and issued publications.

In connection with the approaching International Congress on Tuberculosis, which will be held in Washington, September, 21 to October 12, 1908, a prize of $1,500.00 is offered for the best treatise that may be submitted to that Congress "On the Relation of Atmospheric Air to Tuberculosis."

The treatise may be written in English, French, German, Spanish or Italian. They will be examined and the prize awarded by a Committee appointed by the Secretary of the Smithsonian Institution in conjunction with the Officers of the International Congress on Tuberculosis.

The right is reserved to award no prize if in the judgment of the Committee no contribution is offered of sufficient merit to warrant such action.

The Smithsonian Institution reserves the right to publish the treatise to which the prize is awarded.

Further information, if desired by persons intending to become competitors, will be furnished on application.

CHARLES D WALCOTT,
*Secretary, Smithsonian Institution.*

Washington, February 3, 1908.

---

Two Ages of Men.—There are two periods in a man's life when he is unable to understand women. One is before marriage and the other is after.—*Harper's Weekly.*

---

Sure of Her Ground.—Mistress—"Jane, I saw the milkman kiss you this morning. In the future I will take the milk in."

Jane—"'Twouldn't be no use, mum. He's promised never to kiss anybody but me."—*Illustrated Bits.*

---

Rather Tedious.—Caller—"Do you think the doctor is going to help you, Mr. Jones?"

Jones—"He may, if I can only follow his orders. He told me to drink hot water thirty minutes before each meal, but its hard work to drink hot water for thirty minutes."—*Pittsburg Observer.*

---

Buttermilk.—"Which is the cow that gives the buttermilk?" innocently asked the young lady from the city, who was inspecting the herd with a critical eye.

"Don't make yourself ridiculous," said the young lady who had been in the country before and knew a thing or two. "Goats give buttermilk."—*Springfield Journal.*

---

No Difference.—"Mamma, may I get on the donkey's back?"

"No, dear. But if you are good papa will take you on his back. That will be just the same."—*Rire* (Paris).

